# BCR: a new target in resistance mediated by BCR/ABL-315I?

**DOI:** 10.18632/genesandcancer.93

**Published:** 2016-01

**Authors:** Isabella Haberbosch, Anahita Rafiei, Claudia Oancea, Gerhart Oliver Ottmann, Martin Ruthardt, Afsar Ali Mian

**Affiliations:** ^1^ Department of Hematology, Goethe University, Frankfurt, Germany; ^2^ Deparment of Haematology, School of Medicine, Cardiff University, Cardiff, United Kingdom; ^3^ Cardiff Experimental Cancer Medicine Centre (ECMC), Cardiff, United Kingdom; ^4^ Department of Hematology, University of Zurich, Zurich, Switzerland

**Keywords:** Philadelphia chromosome-positive leukemia, BCR/ABL, resistant mutation T315I, endogenous BCR

## Abstract

Targeting BCR/ABL with Tyrosine kinase inhibitors (TKIs) is a proven concept for the treatment of Philadelphia chromosome-positive (Ph+) leukemias but the “gatekeeper” mutation T315I confers resistance against all approved TKIs, with the only exception of ponatinib, a multi-targeted kinase inhibitor. Besides resistance to TKIs, T315I also confers additional features to the leukemogenic potential of BCR/ABL, involving endogenous BCR. Therefore we studied the role of BCR on BCR/ABL mutants lacking functional domains indispensable for the oncogenic activity of BCR/ABL. We used the factor independent growth of murine myeloid progenitor 32D cells and the transformation of Rat-1 fibroblasts both mediated by BCR/ABL. Here we report that T315I restores the capacity to mediate factor-independent growth and transformation potential of loss-of-function mutants of BCR/ABL. Targeting endogenous Bcr abrogated the capacity of oligomerization deficient mutant of BCR/ABL-T315I to mediate factor independent growth of 32D cells and strongly reduced their transformation potential in Rat-1 cells, as well as led to the up-regulation of mitogen activated protein kinase (MAPK) pathway.

Our data show that the T315I restores the capacity of loss-of-function mutants to transform cells which is dependent on the transphosphorylation of endogenous Bcr, which becomes a putative therapeutic target to overcome resistance by T315I.

## INTRODUCTION

The fine regulation of ABL kinase is lost by its fusion to BCR in the context of the t(9;22). The constitutive activation of the ABL-kinase activity induces aberrant proliferation and neoplastic transformation by constitutive activation of down-stream signaling pathways such as RAS, PI3 kinase, or STATs [1, 2]. Several animal models proved that BCR/ABL is responsible for the induction of the leukemic phenotype related to the t(9;22) [3-6].

The inhibition of aberrant BCR/ABL kinase activity by selective ATP mimetics (TKI) such as imatinib, dasatinib or nilotinib, is a valid concept of causal therapy for Ph+ leukemia [7-11]. Unfortunately in advanced Ph+ leukemia, CML-blast crisis and Ph+ ALL, these TKI select resistant clones, mostly due to the presence of point mutations in BCR/ABL [12, 13]. With the exception of the “gatekeeper” mutation T315I the clinically most relevant mutations which confer resistance against the first generation TKI imatinib are targeted by one of the second generation TKI, dasatinib or nilotinib [14].

The “gatekeeper” mutation T315I confers not only resistance against these ATP-competitors but also against some allosteric inhibitor, such as GNF-2 or against inhibition of oligomerization [15-18]. The only approved exception is the multi-targeted kinase inhibitor ponatinib [19-21]. Other TKIs, such as PF-114, able to target BCR/ABL harboring the T315I are actually in pre-clinical development [6].

The mechanisms by which T315I confers resistance to TKI were mainly attributed to an alteration of the three-dimensional structure of the ATP-binding site, leading to a loss of a fundamental H bond [22-25]. Molecular dynamics simulations on the interactions between BCR/ABL-T315I and imatinib revealed that in addition to the supposedly critical H-bond, also other protein/drug interactions are drastically and unfavorably changed in a sort of “domino effect” primarily induced by the T315I [26]. Additional conformational changes due to the T315I and the other resistance mutants further lead to i.) a steric hindrance for binding the TKIs; ii.) changes in the overall ABL kinase activity and iii.) modified biological activity as compared to the unmutated BCR/ABL [27, 28].

The key event for the activation of the BCR/ABL kinase is the tetramerization mediated by the N-terminal coiled coil (CC) region of the BCR-portion [29, 30]. Accordingly, targeting the CC-domain forces BCR/ABL into a monomeric conformation, abolishes its transformation potential by interfering with its kinase activity and increases sensitivity to imatinib [17, 18, 29, 30]. Tetramerization deficient mutants (ΔCC-BCR/ABL) exhibit decreased TK-activity, resulting in a reduced capacity to mediate factor dependent growth in Ba/F3 cells and the suppression of the transformation potential of BCR/ABL [29, 30].

Noteworthy factor independent growth was suppressed by the inhibition of tetramerization in hematopoietic progenitors expressing BCR/ABL harboring the Y253F and E255K mutations but not the T315I mutation [17, 28]. In fact T315I restores oncogenic activity of BCR/ABL not only in tetramerization deficient but also in other loss-of-function mutants. This biological activity seems to be related to the transphosphorylation of endogenous BCR [28].

In order to further confirm the role of the endogenous BCR for the mediation of resistance mediated by the T315I we investigated further the effect of T315I on the transformation potential and the capacity to mediate factor independent growth of BCR/ABL mutants lacking functional domains in the BCR portion considered indispensable for the oncogenic activity of BCR/ABL.

## RESULTS

### T315I restores capacity to mediate factor independent growth of loss of function mutants of p185*^BCR/ABL^* in 32D cells

Targeting oligomerization either by competitive peptides or by deleting the CC-domain efficiently interferes with the transformation potential of native p185*^BCR/ABL^* but not of p185-T315I^BCR/ABL^. This strongly suggests that the “gatekeeper“ mutation T315I is not only responsible for TKI-resistance, but also confers additional properties to BCR/ABL which is uncovered in the case of a loss of oligomerization.

**Figure 1:**
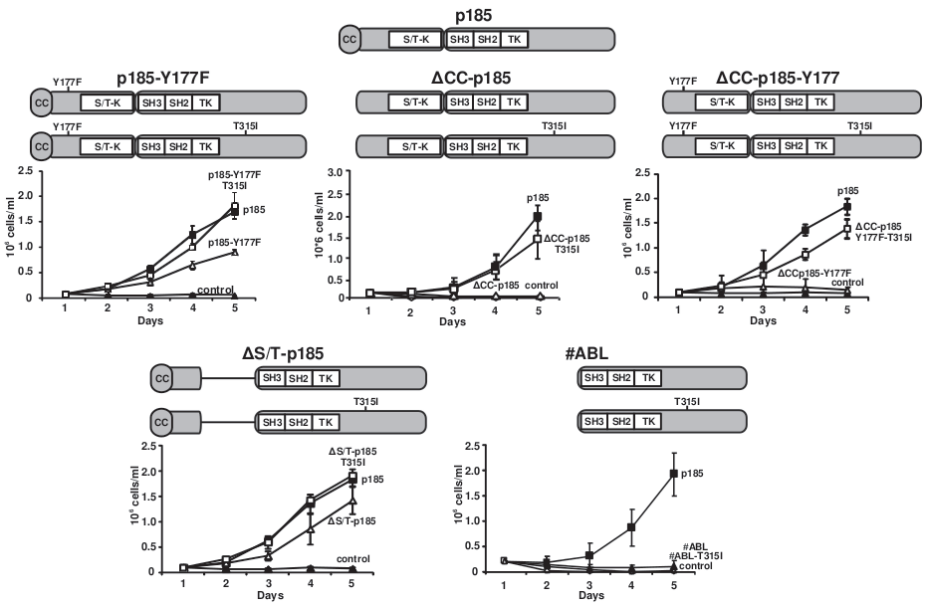
Restoration of factor independent growth of loss of function mutants of p185BCR/ABL by mutation T315I Modular organization of the p185*^BCR/ABL^* mutants+/− T315I. For the determination of factor-independent growth of loss of function mutatnts of p185*^BCR/ABL^* in the presence or absence of T315I mutation, 32D cells were retrovirally transduced with the indicated constructs. The number of viable cells was daily determined by Trypan blue dye-exclusion. The graphs show the means +/− SD of three independent experiments. **A.** p185*^BCR/ABL^* mutants with a point mutation at Y177 (Y177F) +/− T315I. **B.** p185*^BCR/ABL^* mutants lacking the CC oligomerization interface +/−T315I. **C.** p185*^BCR/ABL^* lacking the N-terminal CC-domain together with a point mutation at the Y177 (Y177F) +/−T315I. **D.** ΔS/T-p185 mutants where the N-terminus of BCR comprising the CC-domain and the Y177 phosphorylation site fused to the ABL-portion of the fusion protein +/−T315I. **E.** #ABL - the ABL-portion of the BCR/ABL fusion protein +/− T315I.

To further disclose the influence of T315I on the biology of BCR/ABL we investigated the effects of T315I on “loss of function mutants” of BCR/ABL, unable to mediate factor independent growth of murine hematopoietic progenitors. The schematic representation of the mutants used in this study is shown in Figure 1. We retrovirally infected 32D cells with the constructs and studied their effects on factor independent growth. Native p185^BCR/ABL^ was used as a control.

The phosphorylation at Y177 is indispensable for the function of BCR/ABL. To investigate the effects of T315I on a mutant defective in phosphorylation at Y177 we used p185-T315I-Y177F in which Y177 was mutated to phenylalanine. As shown in Figure 1A, the Y177F reduced factor independent growth of 32D cells mediated by p185^BCR/ABL^, which was completely restored by the presence of T315I.

Next we focused on the influence of T315I on BCR/ABL with deficiencies in oligomerization. As reported in Figure 1B, the presence of T315I restored the capacity of ΔCC-p185 to mediate factor independent growth as shown by the fact that ΔCC-p185-T315I had the identical growth rate as native p185BCR/ABL (Figure 1B).

In order to confirm that both oligomerization interface and Y177 phosphorylation site are dispensable for the function of T315I, we used a combined mutant - ΔCCp185-Y177F - lacking both the CC oligomerization interface and the phosphorylation site at Y177. Even in this mutant, which lacks two functions considered to be indispensable for the activity of BCR/ABL and whose deletion abolished factor independent growth, T315I restored the capacity to confer factor independent growth (Figure 1C).

To disclose the significance of the the serine/threonine (S/T) domain of BCR we investigated the effects of T315I on the factor independent growth of 32D cells expressing a p185*^BCR/ABL^* construct in which the entire S/T domain was deleted with or without the T315I. The deletion of S/T domain attenuated the factor independent growth of 32D cells mediated by p185*^BCR/ABL^*, which was restored by the presence of T315I to the level of native p185*^BCR/ABL^* (Figure 1D).

For the restoration of factor independent growth, T315I seems to need at least parts of the BCR portion as shown by the fact that it was unable to confer factor independence to the ABL-portion of p185*^BCR/ABL^*(#ABL) (Figure 1E).

Taken together, these data indicate that the “gatekeeper” mutation T315I confers additional properties to the loss of function mutants of p185*^BCR/ABL^*, which allow to restore the capacity to mediate factor independence in 32D cells.

### The T315I influences transformation potential of p185*^BCR/ABL^* loss of function mutants

Factor independent growth of 32D cells stands for the substitution of the IL-3 signaling by an alternative survival signal, which can be mediated by activated kinases such as BCR/ABL. To investigate the influence of T315I on the transformation potential of loss-of function mutants of p185^BCR/ABL^, we performed classical transformation assays in order to study both the loss of contact inhibition and anchorage-dependent growth in retrovirally transduced Rat-1 fibroblasts, by focus and colony formation assays, respectively.

**Figure 2:**
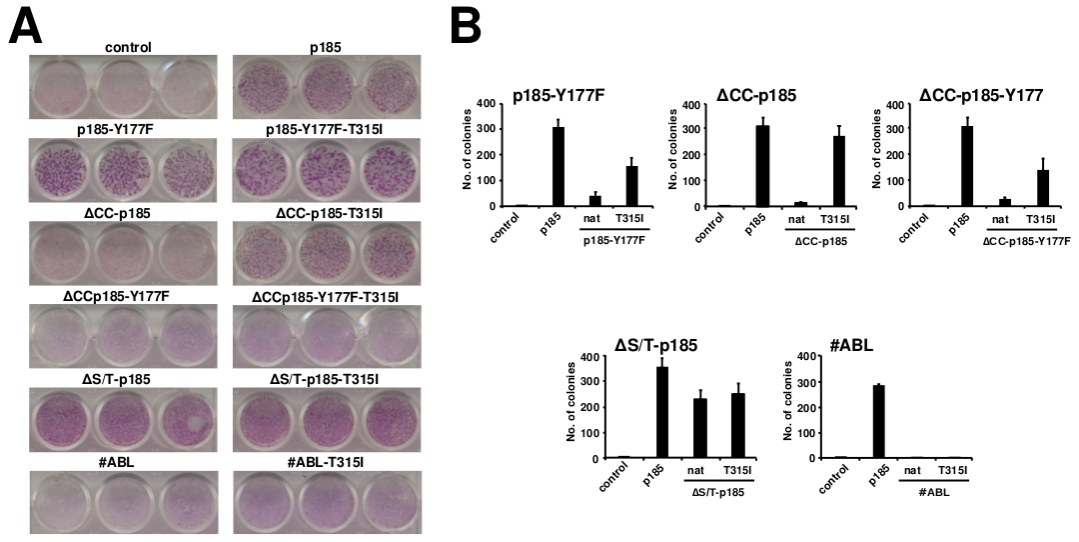
The influence of the resistance mutations on the transformation potential of loss of function mutants of p185BCR/ABL **A.** Focus formation assay - 4×10^4^ infected Rat-1 cells/well were plated in 24-well-plates, grown for 72h to confluence, and incubated for additional 12 days. The plates were then washed, dried, and stained with crystal violet. One representative of each of 3 experiments performed in triplicate is given (34x magnification). **B.** Colony formation - Rat-1 cells were retrovirally transduced with the indicated constructs and seeded at 5×10^3^ cells/well in soft-agar in 6-well-plates. After 15 days, the colonies were counted and the means +/− SDS of triplicates of 2 representative experiments are given.

The abolition of the Y177 phosphorylation site did not influence the lack of contact inhibition of p185*^BCR/ABL^* expressing Rat-1 cells (Figure 2A), but it abolished their ancorage-independent growth, which was restored, even if not completely, by the presence of T315I (Figure 2B).

Targeting oligomerization by the deletion of the CC-domain completely abolished contact inhibition as well as anchorage-dependent growth of p185*^BCR/ABL^*, but both were restored by the T315I as shown by the focus and colony formation (Figures 2A and 2B).

The deletion of S/T alone in the ΔS/T-p185*^BCR/ABL^* in combination with the point mutation Y177F interfered with the transformation potential of p p185*^BCR/ABL^* (Figure 2A and 2B). The T315I did not apparently influence the already full transformation potential of ΔS/T-p185^BCR/ABL^ but “partially” restored colony formation but not focus formation of the ΔS/T-p185-Y177F (Figure 2A and 2B).

As already shown for the factor independent growth T315I was not able to re-establish transformation potential of the isolated #ABL (Figure 2A and 2B).

These data show that T315I is able to influence not only the factor-independent growth of loss of function mutants of p185*^BCR/ABL^*, but also their transformation potential, even if in a different manner as compared to its effect on factor independent growth. Furthermore these data suggest, that the presence of the phosphorylation at Y177 in the BCR-portion of p185*^BCR/ABL^* may play an important role for the capacity of T315I to mediate transformation.

### T315I restores Erk activation by loss of function mutants of p185*^BCR/ABL^*

We have recently shown that the capacity of T315I to restore factor independent growth to the loss of function mutants of p185*^BCR/ABL^* is accompanied by trans-phosphorylation of endogenous BCR at position Y177[28].

**Figure 3:**
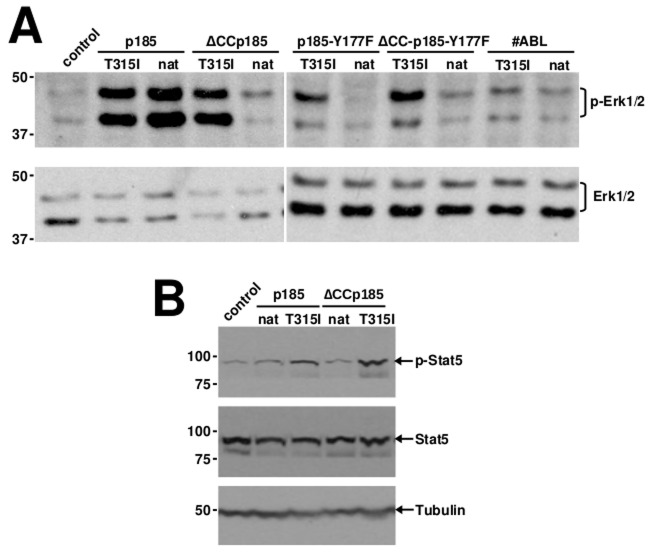
Restoration of aberrant activation of MAPK and Jak/STAT pathways in loss of function mutants of p185BCR/ABL by T315I **A.** Western blotting on lysates of 32D cells expressing the indicated transgenes using antibodies directed against Erk1/2, phosphorylated Erk1/2. **B.** Western blotting on lysates of 32D cells expressing the indicated transgenes using antibodies directed against signal transducer and activator of transcription (STAT5) and phosphorylated STAT5. Molecular mass references (KDa) are given and Tubulin was used as a loading control.

These differences between native p185*^BCR/ABL^* and p185-T315I*^BCRABL^* regarding the apparent importance of the trans-phosphorylation of BCR at Y177 for the resistance conferred by T315I prompted us to investigate the down-stream signaling in the presence or absence of T315I. Therefore, we transduced 32D cells with the indicated constructs. In order to avoid the bias of stress-related activation of signaling pathways these experiments were performed in the presence of IL-3. Erk-activation was assessed by a specific antibody directed against phosphorylated Erk1/2. We found that p185*^BCR/ABL^* and p185-T315I*^BCR/ABL^* fully activated Erk1/2 whereas the loss-of-function was accompanied by the loss of Erk1/2 activation (Figure 3A). T315I restored the Erk1/2 activation in all tested mutants of p185*^BCR/ABL^* with the exception of #ABL, which was not able to exhibit either factor independent growth and or transformation (Figure 1 and Figure 2).

Activation of Stats is indispensable for the transformation potential and the factor independent growth mediated by p185*^BCR/ABL^*. Therefore we studied Stat5-activation in cells expressing p185*^BCR/ABL^* mutants in the presence/absence of the T315I by using a specific antibody directed against phosphorylated Stat5. As shown in Figure 3B, in cells expressing p185*^BCR/ABL^* or p185-T315I*^BCR/ABL^* Stat5 was activated. The oligomerization-deficient ΔCC-p185 was unable to activate, whereas Stat5 was fully activated in cells expressing ΔCC-p185-T315I.

Taken together, these data indicate that the biological effects of the T315I mutation may be mediated through aberrant signaling pathways in addition to trans-phosphorylation of endogenous BCR.

### Endogenous BCR is indispensable for the re-establishment of the factor independent growth and transformation potential of oligomerization deficient ΔCC-p185-T315I

**Figure 4:**
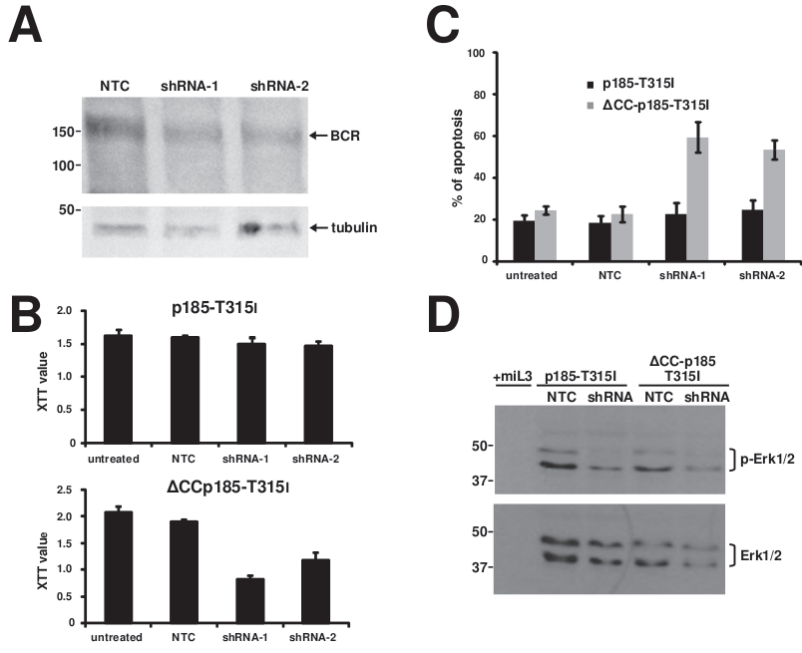
Role of endogenous BCR in the resistance of T315I mutation 32D cells were transduced with the indicated transgenes and retransduced lentivirally with different shRNAs against endogenous BCR. **A.** Western blotting on lysates of 32D cells expressing the indicated transgenes using antibodies directed against BCR. Molecular mass references (KDa) are given and Tubulin was used as a loading control. **B.** For determining cells proliferation, XTT assay was performed. **C.** The apoptosis rate was determined by staining with 7-AAD. The data represent the mean and SD of three independent experiments. **D.** Western blotting on lysates of 32D cells expressing the indicated transgenes using antibodies directed against Erk1/2 and phosphorylated Erk1/2. Molecular mass references (KDa) are given.

In order to further disclose the role of the trans-phosphorylation of endogenous BCR for the p185-T315I^BCR/ABL^ we used RNAi to target BCR. BCR-expression was targeted by specific shRNA in 32D cells expressing either p185-T315I*^BCR/ABL^* or ΔCC-p185-T315I by lentiviral transduction. Efficacy of these shRNA to down-regulate endogenous BCR in these cells in comparison to a non-related control shRNA (NTC) is shown in Figure 4A. Proliferation/cytotoxicity was assessed by a XTT assay. As reported in Figure 4B and 4C, targeting of BCR did not have effect on the factor independent growth of cells expressing p185-T315I*^BCR/ABL^*, whereas it abrogated the capacity of ΔCC-p185-T315I to mediate factor independent growth (Figure 4B and 4C). The high apoptosis rate, measured by 7-AAD-staining, indicated a loss of the capacity to mediate factor independent growth in the absence of endogenous Bcr (Figure 4C).

Next we investigated the possibility to interfere with the role of BCR in the transformation potential of p185-T315I*^BCR/ABL^* or ΔCC-p185-T315I. Thus we performed focus formation assays and colony assays on Rat-1 fibroblasts expressing p185-T315I^BCR/ABL^ or ΔCC-p185-T315I lentivirally transduced with either NTC or shRNA against BCR.

**Figure 5:**
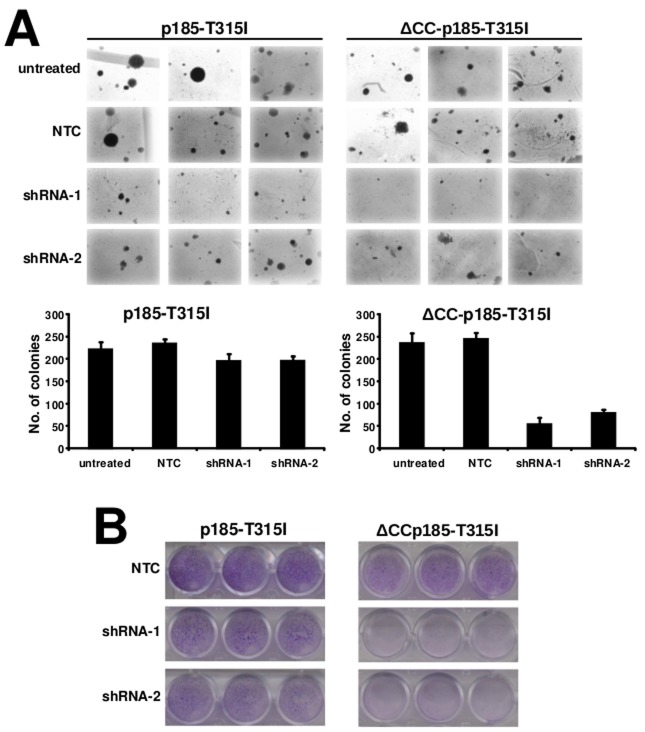
Role of endogenous BCR in the resistance of T315I mutation For the determination for transformation assays, Rat-1 cells were retrovirally transduced with the indicated transgenes and retransduced lentivirally with different shRNAs against endogenous BCR. **A.** Colony formation - Rat-1 cells were retrovirally transduced with the indicated constructs and seeded at 5×10^3^ cells/well in soft-agar in 6-well-plates. After 15 days, the colonies were counted and the means +/− SDS of triplicates of 2 representative experiments are given. **B.** Focus formation assay - 4×10^4^ infected Rat-1 cells/well were plated in 24-well-plates, grown for 72h to confluence, and incubated for additional 12 days. The plates were then washed, dried, and stained with crystal violet. One representative of each of 3 experiments performed in triplicate is given (34x magnification).

As shown in Figures 5A, targeting of BCR attenuated focus and colony formation in Rat-1 cells expressing p185-T315I. In particular this effect was apparent by a reduction of the number but also of the size of the colonies in the semisolid medium. Noteworthy the transformation potential of ΔCC-p185-T315I*^BCR/ABL^* was nearly completely abolished (Figures 5B).

Taken together, these data indicate that targeting endogenous BCR with shRNA strongly reduced the transformation potential p185-T315I*^BCR/ABL^* and ΔCCp185-T315I in Rat-1 cells.

To determine the role of BCR dependent signaling for the survival of cells expressing loss of function mutants of p185*^BCR/ABL^* in the presence of T315I upon factor withdrawal, we studied the effects of targeting BCR on the activation of Erk1/2 and Stat5 in cells expressing either p185-T315I*^BCR/ABL^* or ΔCC-p185-T315I. Here we show that down-regulation of BCR was accompanied by the loss of Erk1/2 activation in both p185-T315I- and ΔCCp185-T315I-positive cells (Figure 4E).

In summary, these data strongly suggest that the endogenous BCR and its related signaling is indispensable for the capacity of T315I to restore transformation potential and the capacity to mediate factor independent growth of loss of function in mutants of p185*^BCR/ABL^*.

## DISCUSSION

The aim of the study was to determine whether and how the the “gatekeeper” mutation T315I confers novel biological features to BCR/ABL influencing its leukaemogenic potential.; (ii) to disclose role of the endogenous BCR for the new biological features determined by T315I in p185*^BCR/ABL^*. We focused our experiments on the p185*^BCR/ABL^* isoform, because it has been shown to behave exactly like the p210*^BCR/ABL^* in our cell model [28, 31, 32].

Ph+ leukemia, neither CML nor Ph+ ALL, ever emerges with a full blown T315I-positive leukemia even if the clone with the mutation is already existing and detectable [33]. Therefore the mutation confers biological features to the clones unveiled by the treatment. One can hypothesize that the presence of the T315I confers a growth disadvantage with respect to native BCR/ABL, which disappears in the moment in which the leukemic cell population driven by native BCR/ABL is suppressed by treatment.

How T315I is modulating the function of BCR/ABL is nearly unknown, mainly due to the fact that actually available models do not clearly reveal differences. Therefore we employed loss of function mutants of native BCR/ABL in which T315I restores at least parts of the BCR/ABL related phenotype.

T315I seems to bypass the autophosphorylation at Y177 as the top of the RAS/ERK-1/2-signalling cascade initiated by BCR/ABL. Its presence leads to an activation of the RAS-signalling even in the absence of a fully functional BCR/ABL kinase. In native BCR/ABL phosphorylation at Y177 provides a docking site for the adapter molecule GRB-2, which stabilizes RAS in its active GTP-bound form by the binding to SOS [34, 35]. Activation of RAS is indispensable for the leukemogenic potential of BCR/ABL [1, 34, 35].

Our data suggest that the transphosphorylation of BCR seen in the presence of the T315I substitutes for the role of the Y177 in BCR/ABL itself and adopted by the endogenous BCR. This is confirmed by our findings that targeting endogenous Bcr by RNAi not only abolished the effects of T315I on Erk1/2 activation, but also the transformation potential and factor independent growth of ΔCC-p185-T315I in murine 32D cells. Translated this scenario into a patient harboring a BCR/ABL-T315I would mean that in the absence of treatment an additional activation of the RAS/MAPK-pathway may also interfere with the proliferation or even senescence [36, 37], whereas it would become relevant only upon treatment, explaining why T315I-positive clones do not grow up in the presence of clones transformed by native BCR/ABL. These different effects on proliferation are confirmed by the fact that the syngeneic CML-like disease induced by BCR/ABL-T315I is significantly delayed as compared to that induced by native BCR/ABL [6].

The mechanism by which the presence of T315I leads to trans-phosphorylation of BCR by BCR/ABL loss of function mutants remains to be clarified. Similar to our findings on p185*^BCR/ABL^*, p210*^BCR/ABL^* transactivates BCR. In fact it co-precipitates BCR from Ph+ cell lines and is a target for the BCR/ABL tyrosine kinase activity [34, 38-41]. BCR/ABL phosphorylates BCR at tyrosine residues which leads to the inhibition of the BCR S/T kinase activity of BCR and thus may not only activate RAS-signaling but also revert the inhibitory effect of S-phosphorylated BCR on the BCR/ABL TK activity [39, 42].

Another important aspect of our study is the fact that we can now distinguish between features important for the mediation of factor independent growth and those important for the classical transformation potential. The combined deletion of the serine/threonine (S/T) and Grb-2 binding (Y177) domains completely abolished the transformation potential of p185 - T315I*^BCR/ABL^* was able to restore only factor independence but not full transformation potential indicating that it needs different domains for different phenotypes like factor independence and transformation potential which might be responsible for the additional oncogenic activity of T315.

In summary, our data show that T315I not only interferes with the affinity of ATP-analogs for the ABL-kinase, but also conferring strong biological activity that seems to be related to the phosphorylation of endogenous bcr. These findings introduce new approaches for the molecular therapy of patients harboring TKI-resistant BCR/ABL mutations.

## MATERIALS AND METHODS

### Plasmids

The cDNAs encoding p185*^BCR/ABL^*, p185-T315, their respective mutants lacking the N-terminal CC domain (ΔCC-p185 and ΔCC-p185-T315I), the ABL part of BCR/ABL (#ABL and #ABL-T315I), deletion mutants of putative BCR serine/threonine kinase domain BCR(1-196)/ABL and BCR(1-196)/ABL-T315), the constructs with a direct fusion of the CC-domain of BCR to the ABL-portion of BCR/ABL (BCC-ABL and BCC-ABL-T315I), the p185-Y177F and p185-Y177F-T315I as well as the ΔCC-p185-Y177F and ΔCC-p185-Y177F-T315I and the related retroviral PINCO vectors have been previously described [28, 30]. Short hairpin (sh) RNA sequences encoding inverted repeats of 21 nucleotides (nt) separated by a 10 nt spacer were purchased from Sigma Aldrich (Steinheim, Germany). The inverted repeats corresponded to 2158-2179bp and 2602-2622bp, of the murine Bcr cDNA. All other constructs have been previously described [18].

### Cell lines

32D cells were obtained from the German Collection of Microorganisms and Cell Cultures (DSMZ, Braunschweig, Germany) and maintained in RPMI-1640 medium supplemented with 10% fetal calf serum (FCS) (Invitrogen) containing 10 ng/ml IL-3 (Cell Concepts, Umkirch, Germany). Ecotropic Phoenix cells, 293T cells and Rat-1 cells were cultured in DMEM supplemented with 10% FCS.

### Transformation assays

Soft-agar and focus formation assays were performed using Rat-1 fibroblasts retrovirally transduced with PINCO vectors harboring native or mutant p185*^BCR/ABL^*. Six-well plates were filled with DMEM supplemented with 10% FCS and 0.5% bacto-agar (DIFCO Laboratories, Detroit, MI, USA). Then, 5×10^3^ transduced Rat-1 cells were suspended in ‘top-agar’ (DMEM supplemented with 10% FCS and 0.25% bacto-agar) and stacked in the wells. Colonies were counted after 15 days incubation. For focus-formation assays in a 24-well plate format, 4×10^4^ transduced Rat-1 cells were plated per well. Foci were stained after 15 days using 1% crystal violet (Sigma-Aldrich).

### Retroviral and lentiviral infection and transfection

Ecotropic retroviral supernatants were obtained as described earlier [30]. Lentiviral supernatant were obtained from 293T cells transfeced with pLKO.1 encoding the desired shRNA, pCMVDR8.91 encoding gag and pol and pMD2.G encoding VSV-G pseudotype envelope protein. 1×10^5^ target cells were plated onto retronectin-coated (Takara-Shuzo, Shiga, Japan) 24-well-plates and exposed to the retroviral or lentiviral supernatant and were centrifuged at 2.200 rpm for 45 min on 32°C. The infection was repeated 3-4 times and infection efficiency was measured after 48 h by determining the percentage of GFP positive cells by flow cytometry.

### Western blotting

Western blot analyses were performed according to widely established protocols. The following antibodies were used: anti-ABL (a-ABL) Santa Cruz Biotechnology, Santa Cruz, CA, USA), anti-phosphorylated ABL specific for the phosphorylated tyrosine-residues 245 (a-p-ABL-Y245) (Upstate-Biotechnology, Lake Placid, NY, USA), anti-BCR (a-BCR) (Santa Cruz Biotechnology), anti-phosphorylated BCR-Y177 (a-p-BCR-Y177) (Cell Signaling, Boston, USA), anti-STAT5, anti phosphorylated STAT5 (Cell Signaling, Boston, MA, USA), anti-Erk1/2 and anti phosphorylated Erk1/2 (Cell Signaling, Boston, MA, USA). Blocking and antibody incubation were performed in 5% low-fat dry milk.

### Proliferation and apoptosis assay

Proliferation was assessed by using the XTT proliferation kit (Roche, Mannheim, Germany), according to the manufacturer's instructions. Apoptosis was measured by 7-AAD staining as described previously [18].
